# Tissue MicroRNA Expression as a Predictor of Response to Immunotherapy in NSCLC Patients

**DOI:** 10.3389/fonc.2020.563613

**Published:** 2021-02-08

**Authors:** Anna Grenda, Paweł Krawczyk, Justyna Błach, Izabela Chmielewska, Tomasz Kubiatowski, Stanisław Kieszko, Kamila Wojas-Krawczyk, Tomasz Kucharczyk, Bożena Jarosz, Iwona Paśnik, Małgorzata Borowiec-Bar, Małgorzata Frąk, Robert Kieszko, Michał Szczyrek, Katarzyna Reszka, Kinga Krukowska, Agnieszka Kolak, Sławomir Mańdziuk, Dariusz Kowalski, Marek Sawicki, Daria Świniuch, Elżbieta Starosławska, Rodryg Ramlau, Justyna Szumiło, Maciej Krzakowski, Janusz Milanowski

**Affiliations:** ^1^ Department of Pneumonology, Oncology and Allergology, Medical University of Lublin, Lublin, Poland; ^2^ Department of Clinical Oncology, Saint John of Dukla Oncology Centre of the Lublin Region, Lublin, Poland; ^3^ Department of Neurosurgery and Paediatric Neurosurgery, Medical University of Lublin, Lublin, Poland; ^4^ Department of Clinical Pathomorphology, Medical University of Lublin, Lublin, Poland; ^5^ Genetics and Immunology Institute of Lublin, Genim LLC, Lublin, Poland; ^6^ Department of Clinical Oncology and Chemotherapy, Medical University of Lublin, Lublin, Poland; ^7^ Department of Lung and Chest Cancer, The Maria Sklodowska-Curie National Research Institute of Oncology in Warsaw, Warsaw, Poland; ^8^ Department of Thoracic Surgery, Medical University of Lublin, Lublin, Poland; ^9^ Department of Oncology, Poznan University of Medical Sciences, Poznań, Poland

**Keywords:** PD-L1, immunotherapy, microRNA, non-small cell lung cancer, SNP, copy number variation

## Abstract

**Introduction:**

Expression of PD-L1 protein on tumor cells, which is so far the only validated predictive factor for immunotherapy, is regulated by epigenetic and genetic factors. Among the most important ones that regulate gene expression are microRNAs.

**Materials and Methods:**

The study included 60 patients with NSCLC who underwent first or second line immunotherapy with pembrolizumab or nivolumab. FFPE materials were collected before the start of immunotherapy. We examined relative expression of microRNAs (miR-141, miR-200a, miR-200b, miR-200c, miR-429, miR-508-3p, miR-1184, miR-1255a) and *PD-L1* mRNA expression. Copy number variation (CNV) of *PD-L1* gene by qPCR and FISH methods were assessed. Two single nucleotide polymorphisms (SNPs) in promoter region of *PD-L1* gene (rs822335 and rs822336) were examined. Expression of PD-L1 protein on tumor cells was assessed by immunohistochemistry (IHC). The response rate to immunotherapy and progression free survival (PFS) measured in weeks and overall survival (OS) measured in months from the start of immunotherapy were evaluated.

**Results:**

Response to immunotherapy was observed in nine patients (15%, including one complete response), disease stabilization in 22 patients (36.7%), and progression in 29 patients (48.3%). Significantly higher (p=0.015) expression of miR-200b and significantly lower (p=0.043) expression of miR-429 were observed in responders compared to patients who did not respond to immunotherapy. The median PFS in the whole group of patients was 16 weeks, and the median OS was 10.5 month. In univariate analysis, the median PFS was significantly higher in patients with high miR-200b expression (HR=0.4253, 95%CI: 0.1737–1.0417, p=0.05) and high miR-508 expression (HR=0.4401, 95%CI: 0.1903–1.0178, p=0.05) and with low expression of miR-429 (HR=0.1288, 95%CI: 0.01727–0.9606, p=0.0456) compared to patients with low and high expression of these molecules, respectively. The median OS was higher in patients with low expression of miR-429 (HR=0,6288, 95%CI: 0,3053–1,2949, p=0.06) compared with patients with high expression of this microRNA. In multivariate analysis, we found that patients with PD-L1 expression on ≥1% of tumor cells compared to patients without PD-L1 expression on cancer cells had a significantly lower risk of progression (HR=0.3857, 95%CI: 0.1612–0.9226, p=0.0323) and death (HR=0.377, 95%CI: 0.1636–0.8688, p=0.022).

**Conclusion:**

The miR-200b and miR-429 molecules in tumor cells seem to have greatest impact on the effectiveness of immunotherapy in NSCLC patients.

## Introduction

Immunotherapy with anti-PD-1 or anti-PD-L1 antibodies has become one of the leading treatment method in patients with advanced non-small cell lung cancer (NSCLC) and small cell lung cancer (SCLC) (1–4).

Predictive factors enabling precise qualification of patients for immunotherapy have not been sufficiently defined, and expression of PD-L1 protein on tumor cells is the only validated factor used in clinical practice in the qualification of NSCLC patients to first line therapy with pembrolizumab (5–8). Unfortunately, only less than 50% of patients with PD-L1 expression on ≥50% of tumor cells respond to first line immunotherapy (9, 10). Reasons for this situation can be sought in the molecular basis of PD-L1 expression.

The variability in the number of copies of the *PD-L1* gene, its polymorphisms, regulatory epigenetic mechanisms, especially microRNA expression in cancer cells, can have a big impact on the expression of PD-L1 protein, and thus on the effectiveness of immunotherapy in patients with various types of cancers (11–15).

In our study, we attempted to correlate genetic and epigenetic factors associated with PD-L1 expression with effectiveness of anti-PD-1 monoclonal antibodies.

## Material and Methods

### Patients

We enrolled (from July 2018 to September 2019) 60 NSCLC patients (41 men and 19 women) with a mean age of 67 years qualified for first or second line immunotherapy with pembrolizumab (n=12, 20%) or nivolumab (n=48, 80%). PD-L1 expression status was assessed in all patients included in the study. Patients received second line immunotherapy regardless of PD-L1 expression on tumor cells if they received chemotherapy in their first-line treatment. First line therapy with pembrolizumab was used only in patients with PD-L1 expression on ≥50% of tumor cells. All patients were in good (n=42, 70%) or very good (n=18, 30%) condition. Fourteen patients (23%) were in stage IIIB and 46 patients (77%) were in stage IV. Adenocarcinoma (AC) was diagnosed in 24 (40%) patients, squamous cell carcinoma (SCC) - in 30 patients (50%), and NSCLC NOS (not-otherwise specified) - in six (10%) patients. **Table 1** shows the demographic and clinical data of our patients.

**Table 1:** Demographic and clinical features of the studied group of patients.

Characteristic	Percentage of tumor cells with PD-L1 expression							
	<50% (n=42, 70%)	≥50% (n=18, 30%)	*p-value*	*Χ^2^*	<1% (n=19, 32%)	≥1% (n=41, 68%)	*p-value*	*Χ^2^*
**Age**								
<65 (n=28)	21 (75)	7 (25)	0.43	0.625	11 (39)	17 (61)	0.23	1.408
≥65 (n=32)	21 (66)	11 (34)			8 (25)	24 (75)		
**Gender**								
Male (n=41)	28 (68)	13 (32)	0.18	0.67	14 (34)	27 (66)	0.54	0.368
Female (n=19)	14 (78)	5 (26)			5 (36)	14 (74)		
**Histological type**								
SqSc (n=30)	**25 (83)**	**5 (17)**	**0.02**	**5.079**	11 (37)	19 (63)	0.40	0.693
AC+NOS (n=24+6 respectively =30)	**17 (57)**	**13 (43)**			8 (27)	22 (73)		
**Stage**								
IIIB (n=14)	**14 (100)**	**0 (0)**	**0.005**	**7.826**	7 (50)	7 (50)	0.09	2.836
IV (n=46)	**28 (61)**	**18 (39)**			12 (26)	34 (74)		
**Smoking status**								
Yes (n=49)	33 (67)	16 (33)	0.34	0.896	17 (35)	32 (65)	0.29	1.132
No (n=11)	9 (82)	2 (18)			2 (18)	9 (82)		
**Response to treatment**								
CR+PR+SD (n=1+8+22)	22 (71)	9 (29)	0.86	0.029	10 (32)	21 (68)	0.92	0.010
PD (29)	20 (69)	9 (31)			9 (31)	20 (69)		

The inclusion criteria for treatment were as follows: age over 18 years, very good or good performance status (PS=0 or 1 according to ECOGS scale), diagnosis of NSCLC (regardless of the pathomorphological type), no mutations in the *EGFR* (*epidermal growth factor receptor*) gene and no rearrangement of the *ALK* (*anaplastic lymphoma kinase*) gene in patients with non-SCC, PD-L1 expression on ≥50% of tumor cells in qualification to first line treatment with pembrolizumab, stage IIIB or IV, presence of measurable neoplastic lesions in computed tomography according to RECIST 1.1 (response evaluation *criteria* in solid tumors), no other contraindications to the use of immunotherapy in accordance with the summary of product characteristic for individual drugs (e.g. autoimmune diseases). Imaging to assess PFS and ORR were performed every 3 months during immunotherapy, and then depending on the clinical situation. In the absence of disease progression after immunotherapy, the computed tomography were continued every 3 months until progression. These criteria were in compliance with the reimbursement regulations in Poland. All patients qualified for immunotherapy who had signed a written consent to participate in the study were included in the study. One hundred twenty-seven patients qualified for immunotherapy were provided with information on the methodology and purpose of the study. The small number of patients results from delays in the reimbursement of immunotherapy in Poland compared to other European Union countries.

We performed a routine examination of PD-L1 expression in formalin-fixed paraffin-embedded (FFPE) material immediately after bronchoscopy and after obtaining the result of a pathomorphological examination. At the same time, material for genetic testing was secured and DNA as well as total RNA was isolated (there were no archival materials). The following factors have been genetically tested:

-relative expression of selected microRNA examined by qPCR (quantitative PCR) method,
-relative mRNA expression of *PD-L1* gene examined by qPCR method,
-copy number of *PD-L1* gene assessed by FISH (fluorescence *in situ* hybridisation) and qPCR methods,
-polymorphisms of the *PD-L1* gene promoter examined by qPCR method,
-protein expression on tumor cells assessed by IHC method (immunochistochemistry).

### RNA Isolation

Total RNA including microRNA was extracted from FFPE tissues using the miRNeasy FFPE Kit (Qiagen Inc., Germany) according to the manufacturers’ instructions. RNA samples were stored at −80°C until synthesis of complementary DNA (cDNA) was performed.

### microRNA Expression

We examined relative expression of microRNAs (miR-141, miR-200a, miR-200b, miR-200c, miR-429, miR-508-3p, miR-1184, miR-1255a) complementary to the 3’UTR region (3’untranslated region) of *PD-L1* mRNA (according to the TargetScan 7.2 and miRBase Sanger). GAPDH (glyceraldehyde-3-phosphate dehydrogenase) and U6 RNA were used as internal control. cDNA was prepared using TaqMan Advanced miRNA cDNA Synthesis Kit (Life Technologies, USA) according to manufacturers’ instructions. cDNA was amplified in real-time PCR performed on Illumina Eco Real-Time PCR System (Illumina Inc, San Diego, USA). The 20 µl of PCR mixture contained: 10 µl of TaqMan Fast Advanced Master Mix, 1 µl of TaqMan Fast Advanced miRNA Assay, 4 µl of RNase free water and 5 µl of cDNA. Reaction conditions were as follows: 95°C for 20 s (enzyme activation) and 40 cycles for 95°C for 5 s and 60°C for 30 s. Ct values were obtained for each examined microRNAs and for internal controls. Analysis was performed using 2^-ΔCt^ method.

### *PD-L1* Messenger RNA (mRNA) Expression

RT-PCR (reverse transcription PCR) for *PD-L1* mRNA was conducted using High-Capacity RNA-to-cDNA Kit (Life Technologies, USA) according to the manufactures’ instructions.

mRNA expression was assessed by using *GAPDH* mRNA as an internal control. Real-time PCR was performed on Illumina Eco Real-Time PCR System (Illumina Inc, San Diego, USA). The qPCR mixture contained: 10 µl of TaqMan Fast Advanced Master Mix (Life Technologies, USA), 1 µl of TaqMan Gene Expression Assay (for PD-L1 or GAPDH separate reactions, Life Technologies, USA), 5 µl of RNaze-free water and 4 µl of cDNA. Reaction was conducted in subsequent conditions: 95°C for 20 s (enzyme activation) and 40 cycles: 95°C for 3 s, 62°C for 30 s. Ct values were obtained for *PD-L1* mRNA and for *GAPDH* mRNA. Analysis was performed using 2^-ΔCt^ method.

### DNA Extraction

DNA was isolated from FFPE tissues using QIAamp DNA FFPE Tissue Kit (Qiagen, Germany) according to the manufactures’ instruction. The quantity and quality of extracted DNA were analyzed using a BioPhotometer UV/Vis Spectrophotometer (Eppendorf, Germany).

### *PD-L1* Promoter Polymorphism (Single Nucleotide Variation - SNV)

Using quantitative real-time PCR, we examined two SNPs of *PD-L1* promoter region: rs822335 (C>T) and rs822336 (C>G). qPCR reaction was performed using 5.5 µl of Genotyping MasterMix (Life Technologies, USA), 4 µl of DNA (5 ng/µl), 0.5 µl of TaqMan SNP Genotyping Assay (for rs822335 and rs822336 separately, Life Technologies, USA). Real-time PCR was performed on Illumina Eco Real-Time PCR System (Illumina Inc, San Diego, USA) in following conditions: initial denaturation and enzyme activation: 95°C for 10 min, and 40 cycles: 95°C for 15 s, 62°C for 90 s.

### CNV of *PD-L1* Gene Assessed by qPCR Method

Copy number variation of *PD-L1* gene were studied using quantitative real-time PCR method based on RNazeP (TaqMan™ Copy Number Reference Assay) as a housekeeping gene. DNA from lymphocytes of sixteen healthy persons were taken as a control. PCR reaction was performed using 5.5 µl of Genotyping MasterMix (Life Technologies, USA), 4 µl of DNA (5ng/μl), 0.5 µl of TaqMan CNV Assay (Life Technologies, USA) on Illumina Eco Real-Time PCR System (Illumina Inc, San Diego, USA) in the following conditions: denaturation and enzyme activation: 95°C for 10 min, and 40 cycles: 95°C for 15 s, 62°C for 90 s. CNV was scored by 2^-ΔΔCt^ method.

### CNV of *PD-L1* Gene Assessed by FISH Method

The ZytoLight SPEC CD274, PDCD1LG2/CEN9 Dual Color Probe (CE-IVD marked, Zytovision, Germany) was used to detect *PD-L1* gene copy number by fluorescence *in situ* hybridization technique. ZytoLight FISH-Tissue Implementation Kit (Zytovision, Germany) was used for pre-staining procedure. For this procedure 3–5 µm FFPE sections were placed on positively-charged glass slides. First, the specimen was kept for 10 min. at 70°C on the hot plate. Slides with samples were then washed twice in xylen for 10 min and dehydrated two times in subsequent solutions of alcohol: in 100% ethanol for 5 min, and in 90% and 70% ethanol for 5 min each. In sequence, the slides were washed twice in deionized water for 2 min and then were immersed for 15 min in pre-warmed Heat Pretreatment Solution Citric at 98°C. Then, the slides were put twice to deionized water for 2 min. After drying, the appropriate amount of pepsin solution was applied on the samples and they were incubated for 12 min at 37°C in a humidity chamber. The slides were put into Wash Buffer for 5 min and were dehydrated in 70%, 90%, and 100% ethanol for 1 min each. After drying, 10 µl of probe mixture was applied to a slide (in the dark) and immediately coverslipped and sealed with rubber cement. The slides were placed for 10 min on hotplate at 75°C and then at 37°C for overnight hybridization. Next day rubber cement was removed, and slides were placed in Wash Buffer at room temperature to allow the coverslips to float off the slides. Afterwards, the slides were washed twice for 5 min in Wash Buffer previously warmed to 37°C. Then they were dehydrated in 70%, 90% and 100% ethanol for 1 min each and allowed to dry in dark room. 10 μl of DAPI counterstaining was applied to the target area, then coverslipped, and the specimens were scored in fluorescence microscope (Nicon Eclipse 55i, Japan).

The SPEC *CD274*, *PDCD1LG2*/CEN 9 Dual Color probe is a mixture of a green fluorochrome direct-labeled probe specific for *CD274* (*PD-L1*) and *PDCD1LG2* (*CD273* or *PD-L2*) genes in chromosome 9 at 9p24.1 and orange fluorochrome direct-labeled probe specific for the classical satellite III region of chromosome 9 centromere. In “healthy” nucleus, two orange and two green signals are expected. In a cell with polysomy or amplification of *PD-L1* and *PD-L2* genes, multiple copies of the green signal or large green signal clusters are observed. The ratio (R) of the number of green signals from the probe complementary to the *PD-L1* gene to the number of red signals from the probe complementary to the centromere was calculated.

At least 60 non-overlapping nuclei was analyzed in each sample in three different regions of interest.

### PD-L1 Protein Expression

Immunohistochemical analyses (IHC) of PD-L1 protein expression were performed on FFPE tissue cut into 3 μm sections and fixed on Thermo Scientific Superfrost Plus™ glass slides. Glass slides with tissue sections were preheated in 59°C on hotplate prior to IHC staining for at least 3 h. PD-L1 protein IHC staining was conducted using VENTANA SP263 antibody on Ventana Benchmark GX equipment according to the manufacturers’ instruction. After staining all glass slides were washed and dehydrated twice in a series of two 96% ethanol and two xylene washing steps, and then coverslipped.

The cut of points for the assessment of cancer cell percentages with PD-L1 expression (<50% and ≥50% of tumor cells with PD-L1 expression or <1% and ≥1% of tumor cells with PD-L1 expression) were adopted from the Updated Analysis of KEYNOTE-024 and KEYNOTE-010 clinical trials, which compared the efficacy of pembrolizumab and first or second line chemotherapy based on platinum compounds or docetaxel (16, 17).

### Statistical Analysis

The response rate to immunotherapy and progression free survival (PFS) measured in weeks as well as overall survival (OS) measured in months from the start of immunotherapy were evaluated. The statistical analysis was made using chi square, U Mann-Whitney, Spearman, Pearson, and Kaplan-Meier tests. Multivariate analysis using Cox proportional hazards regression method with stepwise selection procedures by minimum AIC was used to establish factors affecting patients’ survival. Receiver operating curves (ROC) with area under the curves (AUC) were used to determine the diagnostic value of microRNAs to predict the PFS or OS. The Youden Index has been determined. Analysis were conducted using MedCalc and Statistica softwares.

The study was approved by the Ethics Committee of the Medical University of Lublin, Poland (No. KE-0254/95/2018). In order to collect blood from the patient, we obtained informed consents. The language of informed consents is Polish.

## Results

### Response to Immunotherapy and Molecular Factors

Response to immunotherapy was observed in 9 patients (15%, including one complete response), disease stabilization - in 22 patients (36.7%), and progression - in 29 patients (48.3%). Median PFS in the whole group of patients reached 16 weeks and median OS was 10.5 month.

**Figure 1:**
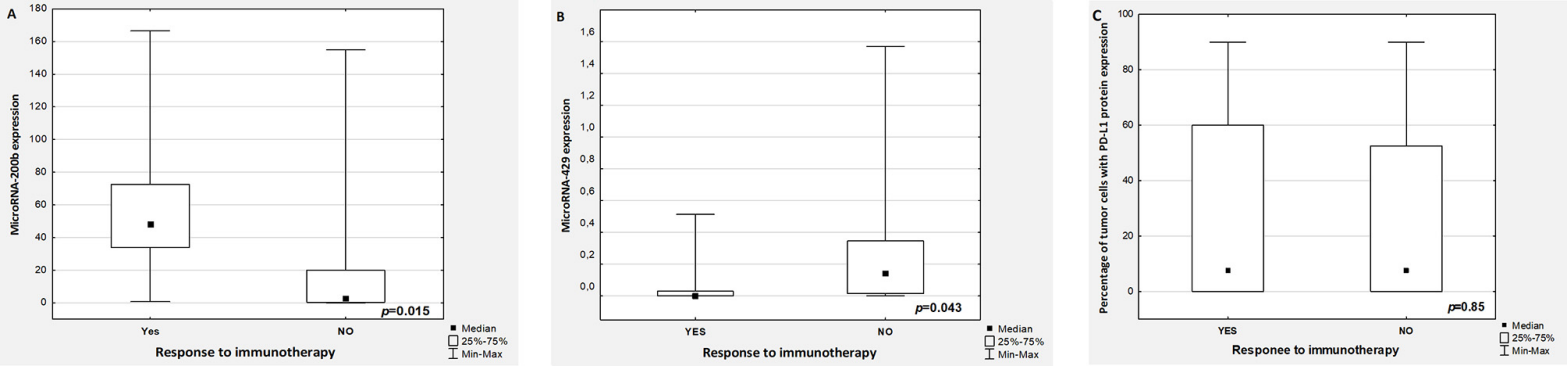
The expression of miR-200b **(A)**, miR-429 **(B),** and percentage of tumor cells with PD-L1 expression **(C)** in patients with and without disease control (YES: stable disease or partial response or complete response, NO: progression disease).

Significantly higher and lower expression of miR-200b and miR-429 respectively, was observed in patients with disease control (p=0.015 and p=0.043 respectively, compared to patients with disease progression (**Figures 1A, B** respectively). There was no differences in percentage of tumor cells with PD-L1 expression in responders and non-responders’ group (p=0.85, **Figure 1C**). The other examined genetic predictive factors, and clinical factors including gender, age, performance status (PS=0 vs. PS=1), stage of disease, pathomorphological diagnosis, line of immunotherapy did not affect treatment response.

**Figure 2:**
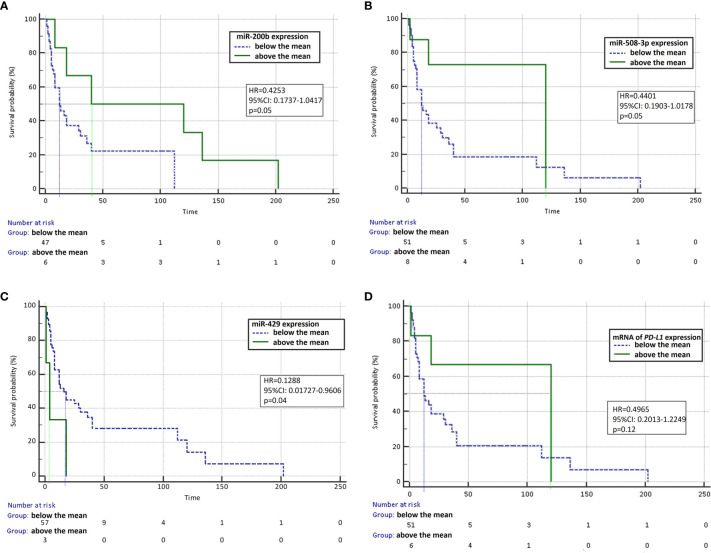
Progression-free survival analysis using Kaplan-Meier test. Survival curves were calculated for patients expressing individual microRNAs and mRNA above and below the median: miR-200b **(A)**, miR-508-3p **(B)**, miR-429 **(C)**, and mRNA of the *PD-L1* gene **(D)**.

In univariate analysis, we observed that the median PFS was significantly higher in patients with high miR-200b expression (HR=0.4253, 95% CI: 0.1737–1.0417, p=0.05, **Figure 2A**) and in patients with high miR-508 expression (HR=0.4401, 95% CI: 0.1903-1.0178, p=0.05, **Figure 2B**) and in patients with low expression of miR-429 (HR=0.1288, 95% CI: 0.01727–0.9606, p=0.04, **Figure 2C**) compared to patients with low and high expression of these molecules, respectively. Moreover, in patients with high mRNA expression of the *PD-L1* gene, the median PFS was not significantly higher than in patients with low mRNA expression for the *PD-L1* gene (HR=0.4965, 95% CI: 0.2013–1.2249, p=0.12, **Figure 2D**). Patients with CC genotype in rs822336 polymorphic site of *PD-L1* gene had insignificantly lower median PFS (HR=0.5330; 95% CI: 0.2473–1.1484; p=0.1) than patients with CG or GG genotypes of this polymorphism. The other examined genetic predictive factors, PD-L1 protein expression on tumor cells and clinical factors did not affect progression free survival of immunotherapy treated patients.

In multivariate analysis with Cox proportional hazards regression method, we found that patients with PD-L1 expression on ≥1% of tumor cells compared to patients without PD-L1 expression on cancer cells had a significantly lower risk of progression (HR=0.3857, 95% CI: 0.1612–0.9226, p=0.0323). Moreover, patients with CC or CG genotypes in rs822336 of *PD-L1* gene as well as with high miR200b expression compared to patients with CC genotype of this polymorphism and with low miR200b expression had an insignificantly lower risk of progression (**Table 2**).

**Table 2:** The factors significantly affected progression free survival and overall survival in patients treated with immunotherapy in multiparameter analysis using Cox proportional hazards regression method.

Factor	p	Hazard ratio	95% CI
**PFS**			
PD-L1 expression on ≥1% of tumor cells	**0.0323**	0.3857	0,1612–0.9226
CG or GG genotype in rs822336 of PD-L1 gene	0.0917	0.3358	0,0945–1.1937
High miRNA-200b expression	0.0771	0.528	0,0190–1.2266
**Overall model fit: χ^2 =^ 4.88, p=0.0272**			
**OS**			
PD-L1 expression on ≥1% of tumor cells	**0.0220**	0.3770	0.1636–0.8688
**Overall model fit: χ ^2 =^ 13.467, p=0.0037**			

Diagnostic value of genetic factors for PFS prediction was calculated in ROC analysis. We found that AUC for miR-200b was 0.848 with specificity of 87% and sensitivity of 67% (95% CI: 0.689–1, p<0.0000, Youden index=0.54), for miR-429 - 0.711 with specificity of 66% and sensitivity of 77% (95% CI:0.413–1, p=0.16, Youden index=0.42), for miR-508-3p −0.674 with specificity of 88% and sensitivity of 73% (95% CI: 0.43–0.918, p=0.16, Youden index=0.42) and for *PD-L1* mRNA - 0 with specificity of 92% and sensitivity of 65% (95% CI: 0.473–1, p=0.07, Youden index=0.59).

In univariate analysis, the median OS was non significantly higher in patients with low expression of miR-429 (HR=0.6288, 95%CI: 0.3053–1.2949, p=0.06) compared with patients with high expression of this microRNA. The median OS in patients treated with pembrolizumab in first-line therapy was not reached, and the differences in death risk reduction between first and second line immunotherapy was not statistically significant (HR=0.7429, 95% CI: 0.3261–1.6923, p=0.4792). An imbalance in the number of patients treated with first and second line of immunotherapy could explain the absence of differences in outcome according to PD-L1 expression. Moreover, most of the patients had metastatic lung cancer (n=46, 77%). This could cause the inability to demonstrate a statistically significant correlation between the stage of the disease and disease outcome. Other examined genetic, immunological, and clinical factors did not influence the median OS according to a univariate analysis.

In multivariate analysis with Cox proportional hazards regression method, we found that patients with PD-L1 expression on ≥1% of tumor cells compared to patients without PD-L1 expression on cancer cells had a significantly lower risk of death (HR=0.377, 95% CI: 0.1636–0.8688, p=0.022, **Table 2**).

### Influence of Molecular Factors on PD-L1 Protein Expression

**Figure 3:**
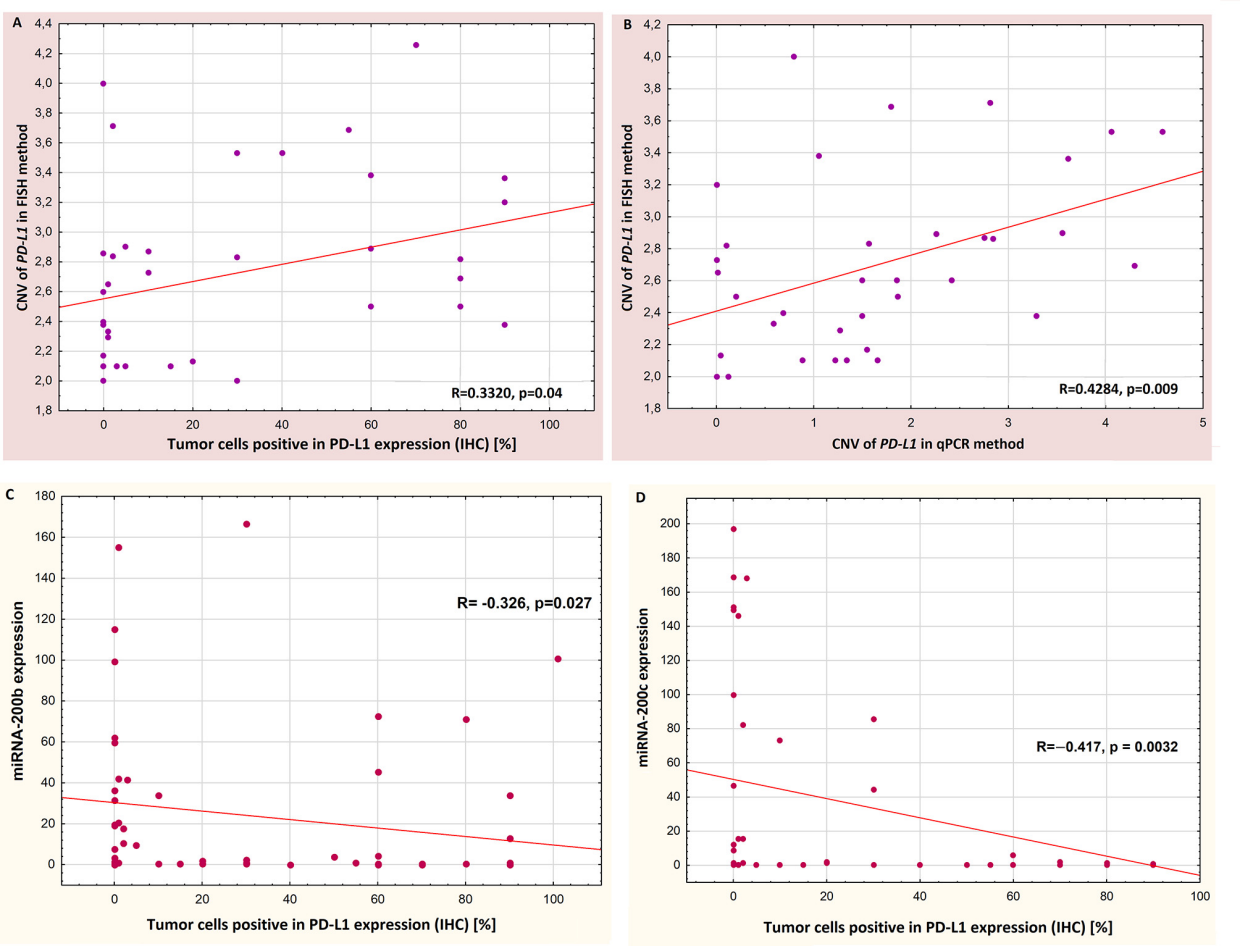
Correlations between: percentage of PD-L1-positive tumor cells and copy number of *PD-L1* gene detected by FISH method **(A)**, copy number of *PD-L1* gene detected by FISH and qPCR methods **(B)**, percentage of PD-L1-positive tumor cells and expression of miR-200b **(C)** and percentage of PD-L1-positive tumor cells and expression of miR-200c **(D)**.

**Figure 4:**
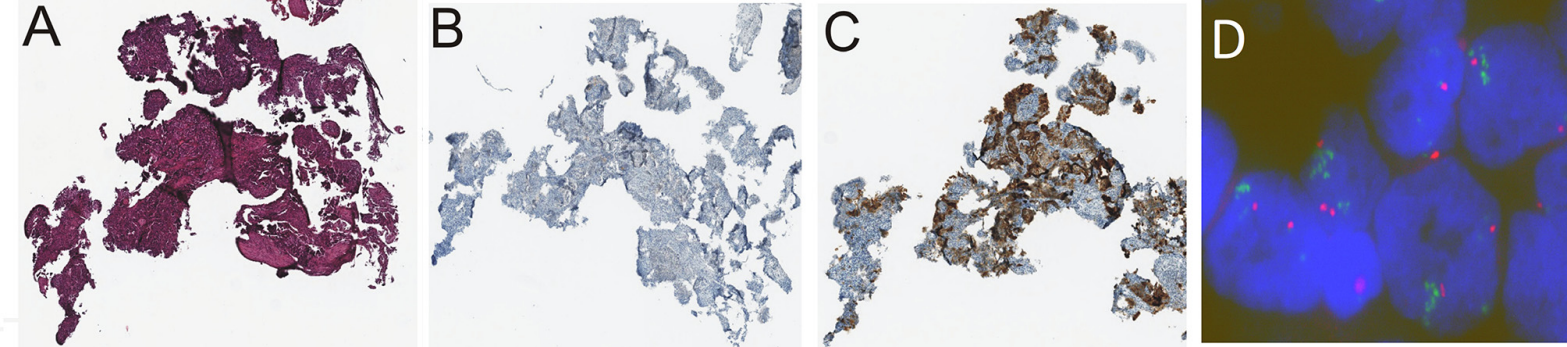
Sample immunohistochemistry (IHC) and FISH staining images taken on the same patient: hematoxylin and eosin staining performed to localize tumor cells **(A)**, negative control of IHC staining **(B)**, IHC staining with the SP263 antibody that detected PD-L1 expression on 70% of tumor cells **(C)**, cell nuclei with visible amplification of the *PD-L1* gene (green); red signals come from the probe complemented to the centromere of chromosome 9; R=4.25 **(D)**.

Percentage of PD-L1-positive cancer cells was significantly correlated with the number of *PD-L1* gene copies in the tumor cells’ nuclei found with the FISH method (Spearman’s R=0.3320, p=0.04, Pearson’s R=0.333,2, p=0.033, **Figure 3A**). There was no correlation between PD-L1 protein and *PD-L1* mRNA expression (p=0.6). Moreover, there was a significant positive correlation between the number of copies of the *PD-L1* gene found in FISH method and PD-L1 gene copies number assessed in qPCR method (Spearman’s R=0.4284, p=0.009, Pearson’s R=0.3388, p=0.014, **Figure 3B**). **Figure 4** shows sample images from the FISH and IHC analysis used to assess *PD-L1* gene copy number and to assess the percentage of tumor cells with PD-L1 protein expression.

The expression of miR-200b and miR-200c significantly negatively correlated with the percentage of tumor cells with expression of PD-L1 protein (R=-0326, p=0.027, Pearson’s R=−0.221, p=0.08 and R=−0.417, p=0.0032, Pearson’s R=0.29, p=0.037, respectively, **Figures 3C, D**).

Patients with CC genotype of the *PD-L1* gene in rs822336 polymorphic site showed significantly lower percentage of tumor cells with PD-L1 protein expression than patients with CG and GG genotype of this polymorphism (p= 0.025).

## Discussion

Based on the KEYTNOTE-024 clinical trial results, the advanced NSCLC patients with ≥50% of PD-L1-positive tumor cells could be treated with pembrolizumab in the first line of therapy. While, locally advanced and advanced patients with PD-L1 expression on ≥1% of tumor cells may be eligible for second line therapy with pembrolizumab (KEYNOTE 010 study). NSCLC patients in stage IIIB and IV, regardless of their PD-L1 expression status, can also be treated in the second line with nivolumab (CheckMate 017 and 057) or atezolizumab (OAK study). Currently, many combination therapies involving immunotherapy and new possibilities for immunotherapy have emerged. Atezolizumab was approved for first line treatment in patients with advanced NSCLC with PD-L1 on ≥50% of tumor cells, and pembrolizumab - in patients with PD-L1 expression on ≥1% of neoplastic cells. Chemotherapy in combination with pembrolizumab in the first line of treatment in patients with advanced NSCLC, regardless of PD-L1 expression on tumor cells, has become common. The combination of nivolumab and ipilimumab is used in the first line of treatment in advanced patients with PD-L1 expression on ≥1% of neoplastic cells (2–4, 16–18).

Tumor PD-L1 expression is still widely used in qualification for immunotherapy. Rapid progression is also observed in patients with PD-L1-positive tumors. On the other hand, treatment response may occur in patients without PD-L1 expression on tumor cells. However, expression of PD-L1 on tumor cells is not an ideal predictive factor for immunotherapy (19).

In our study, we found that there were no differences in the percentage of tumor cells with PD-L1 expression (analyzed as a continuous variable) in patients with disease control and progression occurred during immunotherapy. However, in multivariate analysis, we showed that patients with PD-L1 expression on ≥1% of tumor cells compared to patients without PD-L1 expression on cancer cells had a lower risk of progression and death. In our study the high percentage of patients with squamous cell carcinoma should be explained by the use of bronchoscopic methods used in diagnosis of advanced lung cancer patients. Squamous cell carcinoma is usually a central tumor and the tumor material is easy to collect by bronchoscopy. Moreover, due to the high percentage of smokers in Poland, we still observe a high incidence of squamous cell carcinoma. In a group of 1,923 lung cancer patients diagnosed with bronchoscopy in our clinical center, we found 32.07% patients with squamous cell lung cancer (data not shown in this article).

Previously, clinical studies have been proven that immunotherapy is ineffective in NSCLC patients with *EGFR* gene mutations and *ALK* gene rearrangements. Moreover, the researchers found that high tumor mutation burden (TMB) may be a favorable predictor of immunotherapy in NSCLC patients. Currently, there are many studies to link efficacy of ICIs with presence of abnormalities in different genes, including mutations in *STK11* (*serine-treonine kinase 11*) and *KEAP1* genes. Expression of genes encoding immunomodulatory factors (e.g. cytokines or chemokines) is also considered as a predictive factor for immunotherapy. Many studies devoted to biomarkers that would distinguish hyperprogression an pseudoprogression in patients treated with immunotherapy (20–22). However, only in single studies microRNAs expression is considered as predictor factor for immunotherapy. Investigation on numerous genetic factors that may affect PD-L1 expression are also important.

Therefore, our attention has been focused on microRNAs molecules as potential predictors of response to immunotherapy. In addition, CNV measured by two different methods (qPCR and FISH), assessment of SNPs in the promoter region of the *PD-L1* gene or *PD-L1* mRNA expression were considered as tests of potential utility in qualification to immunotherapy. Our observations show that among mentioned factors profile of microRNAs could identify the patients most likely to benefit from immunotherapy. We tested 8 microRNAs molecules that regulate *PD-L1* mRNA expression according to the TargetScan base.

We found that miR-200b and miR-429 expression could distinguish between NSCLC patients who benefit from immunotherapy and those with disease progression. Their expression and expression of miR-508-3p also influenced the progression free survival in NSCLC patients treated with immunotherapy. On the other hand, there were no differences in the percentage of PD-L1-positive cancer cells in groups of patients with disease control and disease progression. However, the only significant predictive factor which increased the risk of progression or death in a multivariate analysis was PD-L1 expression on ≥1% of tumor cells. Therefore, based on our empirical data, we are joining the opinion that PD-L1 protein expression on tumor cells is not a perfect predictive biomarker for qualification to immunotherapy.

The “microRNAs market” is very wide and a single microRNA molecule has regulatory capacity for dozens or even hundreds of genes. This creates complicated regulatory networks. Therefore, scientific research on these molecules is not easy (23). For example, Tao and colleagues looked for biomarkers of immunotherapy response in patients with prostate cancer (24). They detected that high expression of miR-195 and miR-16 were positively correlated with the biochemical recurrence-free survival of prostate cancer patients. Moreover, the expression of these two molecules were negatively correlated with PD-L1, PD-1, CD80 and CTLA-4 proteins expression (24).

In our study we investigated microRNAs expression in cancer tissue. However, researchers tend to lean toward liquid biopsy in their scientific reports on biomarkers related to the effectiveness of immunotherapy. Boeri and colleagues established plasma immune-related microRNAs-signature classifier (MSC) to identify the risk for an adverse course of the disease in patients with early stages of NSCLC (25). MSC stratified individuals into high, intermediate, and low risk of unfavorable course of the disease. Afterwards, they tested the efficacy of the MSC as prognostic marker in patients with advanced NSCLC treated with nivolumab, pembrolizumab, avelumab, atezolizumab, durvalumab or durvalumab and tremelimumab combination (25). They study included a panel of 24 microRNAs in Custom Taq Array MicroRNA. The study showed that MSC was significantly associated with progression free survival and overall survival. Patients with intermediate and low risk of unfavorable course of the disease estimated based on MSC had higher median of PFS and OS than patients with high risk of disease progression (25). Researchers indicated also that the plasma MSC test could supplement PD-L1 tumor expression test to identify a subgroup of patients with advanced lung cancer who could benefit from immunotherapy. This specific approach using circulating microRNAs profile could be a promising diagnostic tool to assess patients’ chances of responding to immunotherapy.

In our study we also analyzed *PD-L1* mRNA expression. There was no correlation between *PD-L1* mRNA and protein expression. In our opinion, this indicates (which was also pointed out by Wei et al), that PD-L1 expression is subjected to post-transcriptional regulatory mechanisms of microRNAs, protein modification and their transport (26). In this context, we also noted that *PD-L1* mRNA expression, SNP of *PD-L1* gene promoter or CNV of *PD-L1* gene were not a predictor of progression-free survival or overall survival in patients treated with immunotherapy. Unfortunately, the group of patients included in our study was not large and it is limitation of our study. This limitation can be seen especially in the case of subgroups analyzed. The number of patients qualified for our study was due to two problematic aspects. Firstly reimbursement of immune checkpoint inhibitors in NSCLC patients began later in Poland than in other European Union countries. Therefore, we did not manage to collect more patients. Secondly, the number of genetic and immunological tests needed to be performed was large. Therefore, we could only include patients with sufficient tumor materials (in terms of the number and percentage of cancer cells). Due to this limitation of our study, further experiments should be carried out in an enlarged group of patients treated with immunotherapy.

Other researchers also looked at the number of the *PD-L1* gene copies as a predictive marker for immunotherapy. Ikeda examined samples of 94 patients who underwent surgical resection of lung cancer. The authors considered the three copies of the gene as *PD-L1* amplification and they found amplification of *PD-L1* gene in 5% of patients. Also, they noticed the co-amplification of the *PD-L1* gene and the *JAK2* gene in some cases. These genes are located quite close on chromosome 9 (27). Additionally, they tested PD-L1 protein expression on tumor cells by IHC method. No increased expression of PD-L1 protein was found in patients with amplification of the *PD-L1* gene.

Goodman and colleagues examined the number of *PD-L1* gene copies by FISH method in 221 of NSCLC patients. They showed an increase in the number of *PD-L1* gene copies in 11 patients, representing 5% of the study population. In contrast to results obtained by Ikeda et al. (12), all samples with increased *PD-L1* gene copy number had increased expression of PD-L1 protein (≥1% of tumor cells with PD-L1 expression) (12). The results of Goodman’s study are consistent with our results, in which we found a positive correlation between the PD-L1 gene copy number in FISH examination and the percentage of tumor cells with PD-L1 expression in IHC test (12).

Lamberti at al. compared the percentage of PD-L1-positive tumor cells with the results of targeted NGS (next generation sequencing) in large group of 909 non-squamous NSCLC patients (28). They noticed that *PD-L1* gene copy loss is associated with lower response rate and shorter PFS in NSCLC patients treated with immune checkpoint inhibitors. The expression of PD-L1 protein were lower in patients with mutations in the following genes: *STK11, EGFR*, *CTNNB1* (*catenin beta 1*), *APC* (*adenomatous polyposis coli*), and *SMARCA4* (*SWI/SNF related, matrix associated, actin dependent regulator of chromatin, subfamily A, member 4*).

The results of these studies showed that it is legitimate to pay attention to the number of PD-L1 gene copies in NSCLC patients as a predictive factor for immunotherapy. It is also important to examine how *PD-L1* gene CNV and other genetic factors (e.g. genes mutations) affect the expression of PD-L1 protein on tumor cells.

## Conclusion

It seems that evaluation of microRNAs expression in plasma or in tissue of NSCLC patients is a good direction in the search for new predictive factors useful in the qualification of NSCLC patients for immunotherapy. The miR-200b and miR-429 molecules in tumor cells seem to have greatest impact on the effectiveness of immunotherapy in NSCLC patients. However, it should be noted that this is a study involving a small group of patients and further studies on circulating/tissue microRNAs, on a larger group of patients, should be carried out.

## Data Availability Statement

The raw data supporting the conclusions of this article will be made available by the authors, without undue reservation.

## Ethics Statement

The studies involving human participants were reviewed and approved by Ethics Committee of the Medical University of Lublin, Poland. The patients/participants provided their written informed consent to participate in this study.

## Author Contributions

Conception and design: AG, PK, IC, TKuc. Acquisition of data: AG, IC, TKub, SK, DŚ, MB-B, MF, RK, MSz, AK, SM, DK, MSa, ES, RR, JS, MK, JM. Analysis and interpretation of data: AG, KW-K, TKuc, BJ, IP, KR, PK, KK. Drafting the article: AG, PK, TKuc, KW-K. Critically revising: JM, JS, ES, TKub, SM. Final approval of the version to be submitted: AG, PK, JB, IC, TKuc, SK, KW-K, TKub, BJ, IP, DŚ, MB-B, MF, RK, MSz, KR, KK, AK, SM, DK, MSa, ES, RR, JS, MK, JM. All authors contributed to the article and approved the submitted version.

## Conflict of Interest

Authors KR and KK were employed by the company Genim LLC.

The remaining authors declare that the research was conducted in the absence of any commercial or financial relationships that could be construed as a potential conflict of interest.
